# Association Between Syndecan-1, Fluid Overload, and Progressive Acute Kidney Injury After Adult Cardiac Surgery

**DOI:** 10.3389/fmed.2021.648397

**Published:** 2021-07-30

**Authors:** Jiarui Xu, Wuhua Jiang, Yang Li, Haoxuan Li, Xuemei Geng, Xin Chen, Jiachang Hu, Bo Shen, Yimei Wang, Yi Fang, Chunsheng Wang, Zhe Luo, Guowei Tu, Jie Hu, Xiaoqiang Ding, Jie Teng, Xialian Xu

**Affiliations:** ^1^Department of Nephrology, Zhongshan Hospital, Fudan University, Shanghai, China; ^2^Shanghai Institute of Kidney Disease and Dialysis (SIKD), Shanghai Laboratory of Kidney Disease and Dialysis, Shanghai Medical Center of Kidney Disease, Shanghai, China; ^3^Department of Cardiovascular Surgery, Zhongshan Hospital, Fudan University, Shanghai, China; ^4^Department of Critical Care Medicine, Zhongshan Hospital, Fudan University, Shanghai, China; ^5^Department of Nuclear Medicine, Shanghai General Hospital, Jiaotong University, Shanghai, China; ^6^Department of Nephrology, Xiamen Branch, Zhongshan Hospital, Fudan University, Xiamen, China

**Keywords:** syndecan-1, fluid overload, acute kidney injury, cardiac surgery, risk factor

## Abstract

**Background:** Acute kidney injury (AKI) is a common complication after cardiac surgery and the prognosis of AKI worsens with the increase in AKI severity. Syndecan-1(SDC-1) is a biomarker of endothelial glycocalyx degradation. Fluid overload (FO) is associated with poor outcomes in AKI patients and may be related to the damage of endothelial function. This study aimed at demonstrating the association between elevated SDC-1, FO, and AKI progression.

**Methods:** In this prospective study, we screened patients who underwent cardiac surgery and enrolled patients who experienced an AKI within 48 h after surgery from December 1, 2018 to January 31, 2019. Blood and urine samples were collected at the time of AKI diagnosis for plasma SDC-1 (pSDC-1) and urine SDC-1 (uSDC-1) measurements. Fluid balance (FB) = accumulated [fluid intake (L) - fluid output (L)]/body weight (kg) × 100%. FO was defined as FB > 5%. The primary endpoint was progressive AKI, defined as AKI progression from a lower to a higher stage. The patients were divided into progressive AKI group vs. non-progressive AKI group.

**Results:** The quartiles of pSDC-1 concentration (117.3 [67.4, 242.3] ng/mL) showed a graded association with the incidence of progressive AKI, ranging from 5.0, 11.9, 32.6 to 52.4% (*p* for trend < 0.001). Multivariate logistic regression showed that increased pSDC-1 was an independent risk factor for progressive AKI. The AUC-ROC area of pSDC-1 concentration in predicting AKI progression was 0.847. Linear regression showed a positive correlation between FB and pSDC-1 concentration (*R*^2^ = 0.384, *p* < 0.001). In patients with FO, the progressive AKI incidence was significantly higher in the high pSDC-1 (≥117.3 ng/mL) subgroup than in the low pSDC-1 subgroup (58.3 vs. 17.6%, OR = 9.167, *P* = 0.005). In patients without FO, the progressive AKI incidence was also significantly higher in the high pSDC-1 subgroup with a lower odds ratio (30.4 vs. 7.4%, OR = 6.714, *P* = 0.002).

**Conclusion:** Elevated pSDC-1 concentration was associated with progressive AKI after cardiac surgery and showed good predictive ability for progressive AKI. FB was related to the increase of pSDC-1. The interaction between pSDC-1 and FB may further aggravate the progression of AKI.

## Introduction

The incidence of cardiac surgery-associated acute kidney injury (CSA-AKI) varies from 20 to 40% depending on its definition, and the mortality was reported to be between 15 and 30% (1, 2). The prognosis of AKI worsens with the increase in AKI severity. When AKI progresses to stage 3 or renal replacement therapy (RRT), the mortality is as high as 50–80%, and has a higher risk of long-term end-stage renal disease and death (1, 3). Therefore, it is necessary to explore early biomarkers for the prediction of progressive AKI.

Syndecan-1 (SDC-1), a member of the syndecan family, is a protective layer for covering the endothelium (4). It has been demonstrated that SDC-1 is a biomarker for endothelial glycocalyx degradation (5, 6). Rehm et al. provided the first evidence in humans to the shedding of the endothelial glycocalyx during global or regional ischemia/reperfusion (I/R) procedures in major vascular surgery (7). Studies have shown that elevated serum and urinary SDC-1 is a potential biomarker in predicting renal dysfunction and mortality in patients with AKI after cardiac surgery (8, 9).

Fluid overload has been demonstrated as an important risk factor for AKI development and is associated with poor outcomes in critical patients (10, 11). In our previous study, we found that 30-day mortality was significantly higher in the fluid overload group in AKI-RRT patients after cardiac surgery (12). Moreover, it was revealed that in patients who underwent elective surgery and those with severe sepsis, hypervolemia could cause intravascular changes in volume and pressure, thereby destroying the endothelial glycocalyx, with a significant elevation of serum and urinary syndecan-1 (13, 14).

In brief, it is known that SDC-1 can predict AKI occurrence, but little or no studies have demonstrated the association between SDC-1 and AKI progression. It is also unknown whether fluid overload has an effect on this. The aim of this study therefore was to investigate the relationship between glycocalyx degradation (measured as SDC-1), fluid overload, and progressive AKI in adult patients following cardiac surgery, thereby identifying new strategies for the prevention of CSA-AKI.

## Methods

### Patient Selection

**Figure 1:**
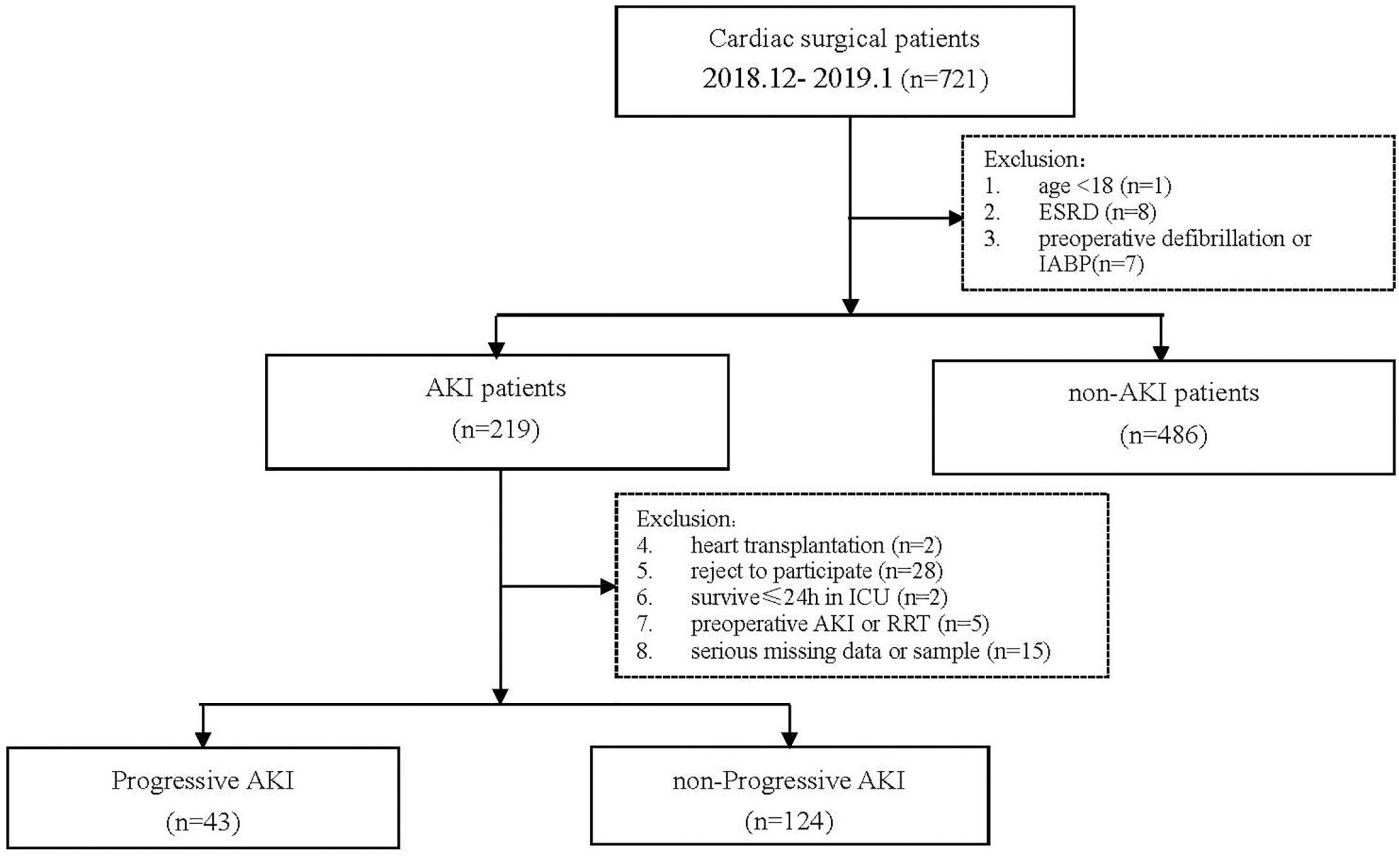
Study flowchart.

This was a single-center, prospective study. We screened 721 patients who underwent cardiac surgery in our hospital from December 1, 2018, to January 31, 2019, and collected data of patients who developed AKI during this period. The exclusion criteria and study flowchart are shown in Figure 1. The Ethical Committee of our hospital approved this study (No. B2017-039).

### Definitions

AKI was defined and graded based on the Kidney Disease Improvement Global Outcomes (KDIGO) 2012 guidelines (15) within 7 days after surgery. Progressive AKI was defined as the worsening of an established AKI stage (16, 17): from AKI stage 1 to stage 2 or 3 or from AKI stage 2–3 during hospitalization. We defined fluid balance (FB) using the equation,

$$\begin{array}{l} FB=\left(fluid\;intake\;\left(L\right)-fluid\;output\;\left(L\right)\right)/admission\;weight\;\left(kg\right)\\ \;\times 100\% \end{array}$$

Fluid overload (FO) was defined as FB > 5% (18).

SCr measured in the ICU was adjusted by the following formula (19–21):

$$\begin{array}{l} \;Adjusted\;SCr=SCr\;\times \;correction\;factor,\\ \text{Where},\;Correction\;factor=[admission\;weight\;(kg)\;\times \;0.6\\ \;+\;cumulative\;(fluid\;intake\;(L)\;-\text{​ }fluid\\ \;output\;(L)]/admission\;weight\;\times \;0.6. \end{array}$$

Complete renal recovery was defined as the ADQI criteria (22): SCr at discharge returning to 50% above baseline SCr.

### Study Design

All patients were admitted to the Intensive Care Unit (ICU) regularly after anesthesia resuscitation. The urine output was recorded every 6 h from the urine collection bag, and fluid intake and output (including fluid removed by drain tube, blood lost from hemorrhage, and urine output) were recorded every day until they were discharged from the ICU. The SCr was monitored every morning. We screened each patient every morning within 48 h after surgery, and AKI was diagnosed according to the KDIGO criteria. Blood and urine samples of the AKI patients were collected at the time of AKI diagnosis for the SDC-1 measurements, and the cumulative fluid balance was calculated from the time of operation to the time of AKI diagnosis.

The main endpoint was progressive AKI. The secondary endpoints were RRT rate, complete renal recovery rate, 28-d mortality, in-hospital mortality, length of ICU stay and hospital stay.

### Measurement of Syndecan-1

Fresh blood and urine samples were centrifuged at 3,000 rpm for 10 min, after which the separate plasma and urine supernatant were collected and stored at −80°C. The pSDC-1 and uSDC-1 concentrations were measured within 6 months after surgery, using sCD138 ELISA kits (Human Syndecan 1, Abcam) according to the manufacturer's instructions (13, 23, 24). SDC-1 is also known as CD138, and soluble CD138 (sCD138) means SDC-1 in the circulation or body fluid. The detection range was 8–256 ng/mL, and the coefficient of variation was 6.2%.

### Statistical Analysis

To estimate the sample size for this study, we applied our unpublished data from a pre-test of 20 AKI patients for the pSDC-1 concentration showing a 2-fold increase in patients with progressive AKI compared with those who did not progress. The coefficient of variation of pSDC-1 was 0.800. Based on these parameters and taking into account the design effect of consecutive sampling (1.5-fold), a minimum of 35 patients was needed to achieve a 90% power and significance level (alpha) of 0.05 in detecting a 2-fold increase in pSDC-1 concentration in progressive AKI patients.

Continuous variables were expressed as mean ± SD or median and interquartile range, and were compared using independent sample *t*-test or Mann-Whitney test. Categorical variables were expressed as proportions and compared using the chi-square or Fisher exact test. *P* < 0.05 were considered statistically significant. The correlation between fluid balance and syndecan-1 concentrations was assessed using linear regression. We included the variables with a *p* < 0.10 from the baseline characteristics table into the univariate regression analyses to identify risk factors for AKI progression, and those with *p* < 0.05 were further included in the multivariate analysis. The area under the curve receiver operating characteristic (AUC-ROC) was used to analyze the predictive value of syndecan-1 for AKI progression. SPSS 22.0 software (IBM Corporation, Armonk, NY, USA) was used for overall statistical analysis.

## Results

### Baseline Characteristics

Finally, 167 AKI patients were enrolled in the analysis, including 43 (25.7%) in the progressive AKI group and 124 (74.3%) in the non-progressive AKI group. The preoperative and intraoperative conditions of the two groups are shown in Table 1. There were no significant differences in gender, age, or weight between the two groups. The body mass index (BMI) in the progressive AKI group was significantly lower than that in the non-progressive AKI group. The estimated glomerular filtration rate (eGFR) in the progressive AKI group was significantly lower than that in the non-progressive AKI group. The type of surgery was comparable between the two groups. The CPB duration and aortic clamping duration in the progressive AKI group were significantly longer than in the non-progressive AKI group.

**Table 1:** Baseline characteristics in progressive AKI vs. non-progressive AKI groups.

	**All**	**Non-progressive AKI group**	**Progressive AKI group**	***P***
	***n* = 167**	***n* = 124**	***n* = 43**	
Preoperative				
Male [*n* (%)]	117 (70.1%)	89 (71.8%)	28 (65.1%)	0.411
Age	60 ± 12	59 ± 12	61 ± 13	0.358
Weight (kg)	69.3 ± 13.5	69.9 ± 14.0	67.5 ± 11.9	0.316
BMI(kg/m^2^)	24.8 ± 3.9	25.1 ± 4.0	23.8 ± 3.4	0.058
Hypertension [*n* (%)]	81 (48.5%)	56 (45.2%)	25 (58.1%)	0.142
Diabetes [*n* (%)]	15 (9.0%)	11 (8.9%)	4 (9.3%)	0.932
History of cardiac surgery [*n* (%)]	17 (10.2%)	14 (11.3%)	3 (7.0%)	0.420
NYHA>II [*n* (%)]	80 (47.9%)	61 (49.2%)	19 (44.2%)	0.571
Coronary angiography[*n* (%)]	102 (61.1%)	72 (58.1%)	30 (69.8%)	0.175
Contrast medium (ml)	57 ± 19	53 ± 14	69 ± 27	0.002
BUN(mmol/L)	7.8 ± 3.1	7.9 ± 3.3	7.6 ± 2.6	0.589
SCr(μmol/L)	96.6 ± 30.2	94.8 ± 28.3	102.4 ± 34.9	0.179
eGFR[ml/(min/1.73 m^2^)]	77.6 ± 20.6	79.6 ± 20.7	70.5 ± 17.9	0.009
eGFR <60 ml [*n* (%)]	33 (19.8%)	19 (15.3%)	13 (30.2%)	0.032
Albumin (g/L)	39.6 ± 5.7	39.4 ± 6.1	40.3 ± 4.2	0.372
Proteinuria [*n* (%)]	22 (13.2%)	14 (11.3%)	8 (18.6%)	0.221
Intra-operative				
Type of surgery				0.262
- Valve	81 (48.5%)	64 (51.6%)	18 (41.9%)	
- coronary artery bypass graft	29 (17.4%)	23 (18.5%)	6 (13.9%)	
- large vessels	36 (21.6%)	24 (19.4%)	12 (27.9%)	
- Others	21 (12.6%)	13 (10.5%)	8 (18.6%)	
Use of CPB [*n* (%)]	145 (86.8%)	104 (83.9%)	41 (95.3%)	0.055
CPB duration (min)	128 ± 75	112 ± 65	175 ± 84	<0.001
Aortic clamping duration (min)	71 ± 38	66 ± 37	87 ± 38	0.002
Ultrafiltration volume (ml)	2,628 ± 1,492	2,413 ± 1,436	3,247 ± 1,492	0.001

*BMI, body mass index; NYHA, New York Heart Association; BUN, blood urea nitrogen; SCr, serum creatinine; eGFR, estimated glomerular filtration rate; CPB, cardiopulmonary bypass; FB, fluid balance*.

### Postoperative Variables and Short-Term Outcomes

The interquartile pSDC-1 and uSDC-1 concentrations in all patients were 117.3 [67.4, 242.3] ng/mL and 54.2 [34.9, 110.2] ng/mL, respectively. The pSDC-1 concentration in the progressive AKI group was significantly higher than that in the non-progressive AKI group (245.2[78.9, 290.5] vs. 96.6[51.4, 150.6] ng/mL, *p* < 0.001). There were no significant differences in uSDC-1 concentration between the two groups (86.8 [42.3, 148.7] vs. 52.1 [22.3, 108.9] ng/mL, *p* = 0.193). The accumulated FB in the progressive AKI group was significantly higher (6.0 ± 3.6 vs. 2.5 ± 1.8, *p* < 0.001). The lactic acid and procalcitonin (PCT) levels in the progressive AKI group were significantly higher as well (Table 2).

**Table 2:** Postoperative variables and shot-term outcomes in progressive AKI vs. non-progressive AKI groups.

	**All**	**Non-progressive AKI group**	**Progressive AKI group**	***P***
	***n* = 167**	***n* = 124**	***n* = 43**	
Postoperative (ICU admission)				
APACHE II score	11.4 ± 5.5	10.2 ± 4.9	15.0 ± 5.7	<0.001
Euro Score	4.7 ± 2.7	4.5 ± 2.6	5.3 ± 2.8	0.091
Postoperative (at AKI diagnosis)				
AKI stage (initial)				0.467
- Stage 1	135 (80.8%)	102 (82.3%)	33 (76.7%)	
- Stage 2	24 (14.4%)	16 (12.9%)	8 (18.6%)	
- Stage 3	8 (4.8%)	6 (4.8%)	2 (4.7%)	
pSDC-1**(**ng/ml**)**	117.3 [67.4, 242.3]	96.6 [51.4, 150.6]	245.2 [78.9, 290.5]	<0.001
uSDC-1(ng/ml)	54.2 [34.9, 110.2]	52.1 [22.3, 108.9]	86.8 [42.3, 148.7]	0.193
FB(%)	3.4 ± 2.8	2.5 ± 1.8	6.0 ± 3.6	<0.001
Fluid overload [n(%)]	53 (31.7%)	28 (22.6%)	25 (58.1%)	<0.001
Lactic acid (mmol/L)	4.3 ± 3.5	3.5 ± 2.4	6.6 ± 4.8	<0.001
PCT (ng/ml)	1.3 [0.5, 4.9]	1.1 [0.4, 4.2]	3.9 [2.7, 7.8]	0.006
Outcomes				
AKI stage (worst)				<0.001
- Stage 1	102 (61.1%)	102 (82.3%)	0	
- Stage 2	47 (28.1%)	16 (12.9%)	31 (72.1%)	
- Stage 3	18 (10.8%)	6 (4.8%)	12 (27.9%)	
RRT [*n*(%)]	17 (10.2%)	6 (4.8%)	11 (25.6%)	<0.001
Complete renal recovery [*n*(%)]	133 (79.6%)	104 (83.9%)	29 (67.4%)	0.021
Length of hospital stay (d)	17 ± 8	15 ± 7	21 ± 9	<0.001
Length of ICU stay (d)	4 [2, 6]	3 [2, 4]	5 [4, 16]	<0.001
Mechanical ventilation days	2 [1, 3]	2 [1, 3]	3 [2, 10]	0.003
In-hospital mortality [*n* (%)]	6 (3.6%)	1 (0.8%)	5 (11.6%)	0.001
28-d mortality [*n* (%)]	4 (2.4%)	1 (0.8%)	3 (7.0%)	0.022

*APACHE, acute physiology and chronic health evaluation; AKI, acute kidney injury; FB, fluid balance; PCT, procalcitonin; RRT, renal replacement treatment; ICU, intensive care unit*.

In the progressive AKI group, there were 8 patients with stage 2 progressed to stage 3, and 31 patients with stage 1 progressed to stage 2, and 2 patients with stage 1 progressed to stage 3. Significantly more patients received RRT treatment in the progressive AKI group (25.6 vs. 4.8%, *p* < 0.001). The length of hospital and ICU stay, and mechanical ventilation duration in the progressive AKI group were significantly longer than in the non-progressive AKI group. The 28-d mortality and in-hospital mortality was significantly higher in the progressive AKI group than in the non-progressive AKI group (7.0 vs. 0.8%, *p* = 0.022; 11.6 vs. 0.8%, *p* = 0.001) (Table 2).

### Syndecan-1 and Progressive AKI

**Figure 2:**
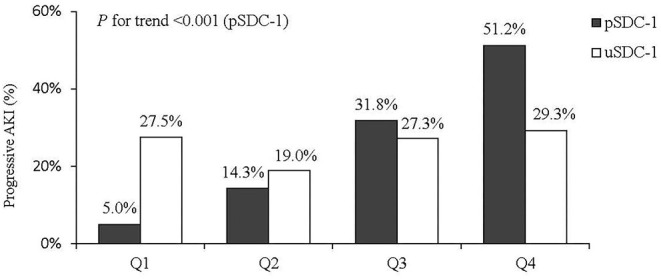
Quartiles of pSDC-1 and uSDC-1 concentration and AKI progression.

Associations between progressive AKI and the pSDC-1 and uSDC-1 concentrations were categorized into quartiles (Figure 2). The quartiles of pSDC-1 concentration (117.3 [67.4, 242.3] ng/mL) showed a graded association with the incidence of progressive AKI, ranging from 5.0, 11.9, 32.6 to 52.4% (*P* for trend < 0.001). We divided patients into high pSDC-1 group (*n* = 82) and low pSDC-1 group (*n* = 85) according to the median (117.3 ng/mL). The progressive AKI incidence in the high pSDC-1 group was greater than that in the low pSDC-1 group (42.7 vs. 9.4%, *p* < 0.001).

### Logistic Regression Analysis for the Risk Factors of Progressive AKI

The univariate logistic regression showed that baseline eGFR <60 mL/min/1.73 m^2^, increased CPB duration, aortic clamping duration, intraoperative ultrafiltration volume, APACHE II score, Euro Score, pSDC-1 concentration, fluid overload, lactic acid levels, and PCT were risk factors for progressive AKI (Table 3). In multivariate logistic regression, the independent risk factors for progressive AKI included increased CPB duration (OR = 1.014, 95% CI: 1.002–1.046), increased APACHE II on ICU admission (OR = 1.201, 95% CI: 1.013–1.437), and increased pSDC-1 concentration (OR = 1.030, 95% CI: 1.001–1.068) (Table 3).

**Table 3:** Logistic regression analyze the risk factors for AKI progression.

	**OR (*95%CI*)**	***P***
Univariate		
BMI	0.908 (0.787–1.047)	0.183
Contrast medium	1.025 (0.997–1.054)	0.086
Baseline eGFR <60 ml/(min/1.73 m^2^)	1.886 (0.941–3.944)	0.016
CPB duration	1.018 (1.007–1.030)	0.002
Aortic clamping duration	1.033 (1.012–1.054)	0.002
Intraoperative ultrafiltration volume	1.001 (1.000–1.001)	0.039
APACHE II score	1.209 (1.083–1.350)	0.001
Euro score	1.067 (0.891–1.277)	0.480
pSDC-1	1.012 (1.006–1.018)	<0.001
Fluid overload (Y/N)	4.200 (1.350–13.065)	0.013
Lactic acid (mmol/L)	1.274 (1.087–1.493)	0.003
PCT (ng/ml)	1.064 (0.998–1.133)	0.056
Multivariate		
CPB duration	1.014 (1.002–1.046)	0.026
APACHE II on ICU admission	1.201 (1.013–1.437)	0.046
pSDC-1	1.030 (1.001–1.068)	0.020

*BMI, body mass index; NYHA, New York Heart Association; eGFR, estimated glomerular filtration rate; APACHE, Acute Physiology and Chronic Health Evaluation; PCT, procalcitonin*.

The AUC-ROC areas for increased pSDC-1 concentration to predict AKI progression and 28-d mortality were 0.847 and 0.892. The cut-off values were 123.8 ng/ml (with the sensitivity 0.897 and the specificity 0.652) and 277.2 ng/ml (with the sensitivity 0.667 and the specificity 0.883), respectively.

### Syndecan-1 and Fluid Overload

**Figure 3:**
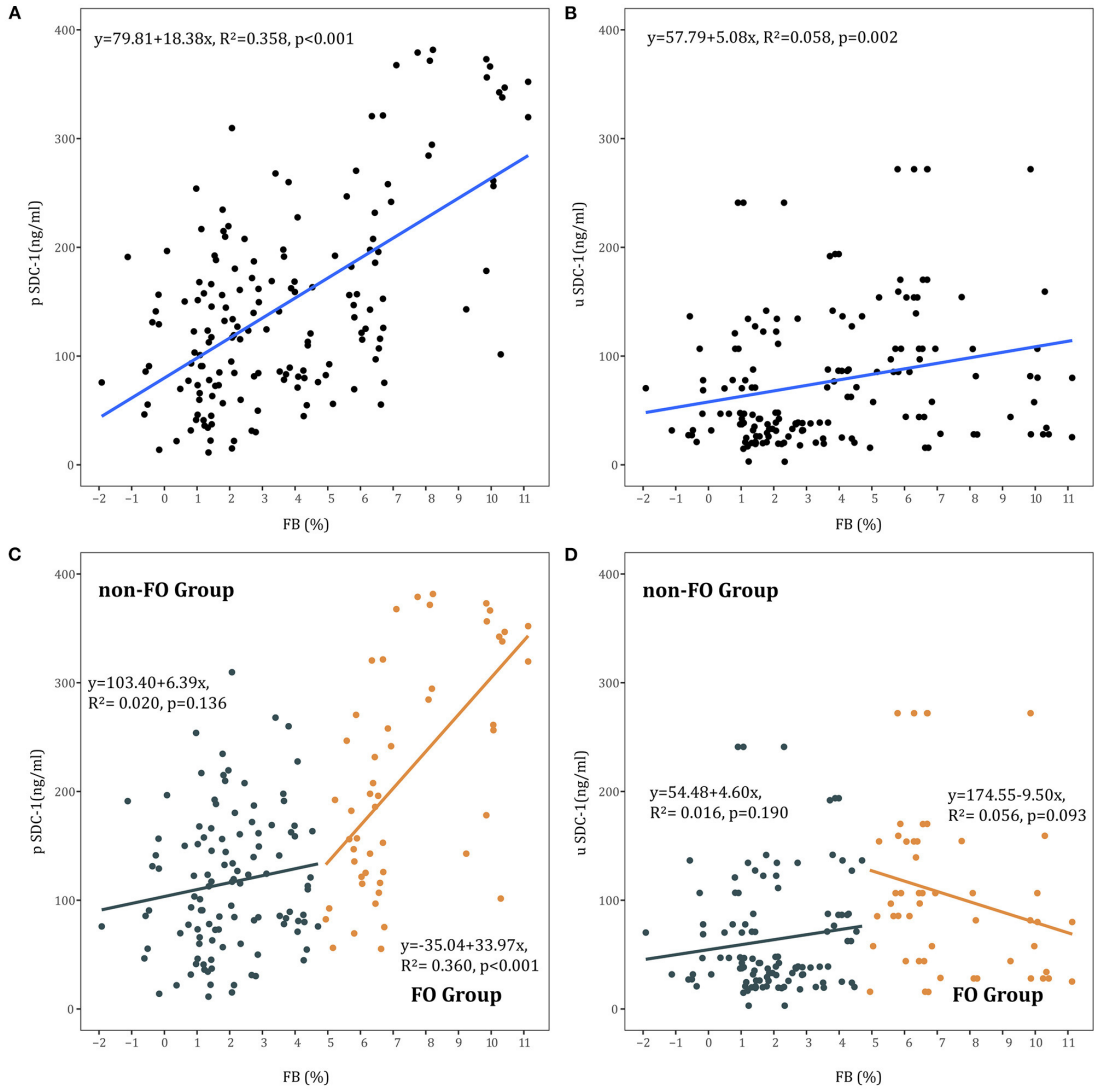
**(A)** Association between FB and pSDC-1 in all AKI patients. **(B)** Association between FB and uSDC-1 in all AKI patients. **(C)** Association between FB and pSDC-1 in non-FO group and FO groups. **(D)** Association between FB and uSDC-1 in non-FO group and FO groups.

Linear regression showed a positive correlation between FB and pSDC-1 concentration in all AKI patients (*R*^2^ = 0.358, *p* < 0.001) and in the FO group (*R*^2^ = 0.360, *p* < 0.001). In the non-FO group, there was no significant correlation between FB and pSDC-1 concentration (*R*^2^ = 0.020, *p* = 0.136) (Figures 3A,C).

Moreover, linear regression showed a positive correlation between FB and uSDC-1 concentration in all AKI patients (*R*^2^ = 0.058, *p* = 0.002). There was no correlation between FB and uSDC-1 concentration in the FO and non-FO groups, respectively (Figures 3B,D).

We further performed a logistic regression analysis to understand the potential risk factors for high pSDC-1 (≥117.3 ng/mL). It was showed that FB was the only risk factor for high pSDC-1 (OR = 1.531, 95% CI: 1.141–2.054) (Table 4).

**Table 4:** Logistic regression analyze the risk factors for High pSDC-1 (≥117.3 ng/ml).

	**OR (*95%CI*)**	***P***
Univariate		
Baseline eGFR [ml/(min/1.73 m^2^)]	0.996 (0.984–1.021)	0.531
CPB duration	1.012 (1.004–1.020)	0.004
Aortic clamping duration	1.009 (0.997–1.016)	0.125
APACHE II score	1.124 (1.026–1.232)	0.012
Euro score	1.160 (0.979–1.376)	0.087
FB (%)	1.586 (1.258–2.010)	<0.001
Lactic acid (mmol/L)	1.174 (1.005–1.371)	0.043
PCT (ng/ml)	1.112 (1.006–1.254)	0.048
Multivariate		
FB (%)	1.531 (1.141–2.054)	0.005

*eGFR, estimated glomerular filtration rate; APACHE, Acute Physiology and Chronic Health Evaluation; FB, fluid balance; PCT, procalcitonin*.

### Syndecan-1, Fluid Overload, and Progressive AKI

**Figure 4:**
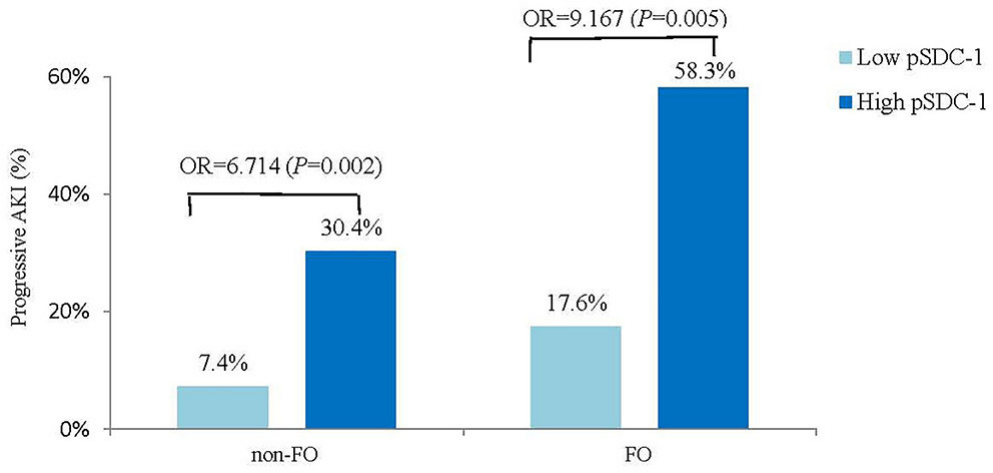
Compare the progressive AKI incidence between high vs. low pSDC-1 in non-FO vs. FO groups.

The progressive AKI incidence was highest in the FO & high pSDC-1 group (58.3%, *n* = 21), followed by the non-FO & high pSDC-1 group (30.4%, *n* = 14), FO & low pSDC-1 group (17.6%, *n* = 3), and non-FO & low pSDC-1 group (7.4%, *n* = 5) (Figure 4).

In the FO group, the progressive AKI incidence was significantly higher in the high pSDC-1 subgroup than in the low pSDC-1 subgroup (58.3 vs. 17.6%, OR = 9.167, *p* = 0.005). In the non-FO group, the progressive AKI incidence was also significantly higher in the high pSDC-1 subgroup with a lower odds ratio (30.4 vs. 7.4%, OR = 6.714, *p* = 0.002) (Figure 4).

## Discussion

In this study, we found that elevated pSDC-1 concentration measured at AKI diagnosis was associated with progressive AKI after cardiac surgery. Moreover, pSDC-1 alone showed a good predictive ability for progressive AKI with an AUC-ROC of 0.847. We also found a positive correlation between FB and pSDC-1 concentration, and the interaction between pSDC-1 and FB may further aggravate AKI, as the odds ratio of elevated pSDC-1 for progressive AKI was higher in the FO group than in the non-FO group.

Our findings had two possible explanations. First, cardiac surgery-induced I/R injury results in damage to the endothelial glycocalyx and increased SDC-1 (25), and it has been demonstrated that there is a relationship between the severity of I/R injury and the extent of endothelial activation (26). In our previous findings in mice, I/R induced shedding of SDC-1 from the kidney resulted in the elevation of serum SDC-1 level (27). Therefore, our assumption was that for AKI patients, persistent I/R injury may continue to increase the pSDC-1 concentration. This may be why the pSDC-1 concentration in the progressive AKI group was significantly higher than that in the non-progressive AKI group. Inflammation also plays an important role. Our study found in the univariate logistic regression (Table 4) that PCT concentration was one of the risk factors for elevated pSDC-1. CPB induces a systemic inflammatory reaction, and inflammatory cytokines (IL-6, IL-8, and IL-10) were associated with glycocalyx degradation, measured as plasma syndecan-1 concentrations (28). As endothelial cells serve as the first barrier to prevent inflammation, SDC-1 degradation results in increased vessel wall permeability, enhanced adhesion of leukocytes, and increased perivascular inflammation (4, 5, 29). Thus, the interaction between inflammation and SDC-1 may lead to a vicious cycle, which aggravates the pathological process in the renal.

This confirms that positive fluid balance is associated with worse outcomes (10–12). Patients are prone to receive fluid resuscitation after cardiac surgeries due to hemodynamic instability or low cardiac output syndrome. In the present study, we found a positive correlation between fluid balance, and pSDC-1 and uSDC-1 in AKI patients. SDC-1 contributed to maintaining cell shape and structure by regulating the integrin and tight junction proteins (Occludin and ZO-1) (30, 31). Hypervolemia may stretch the vascular wall and worsen vascular permeability, possibly by atrial natriuretic peptide-induced damage to the glycocalyx (32). Chappel (13) and Puskarich (14) found that in patients who received elective surgery and those with severe sepsis, hypervolemia increased syndecan-1. Furthermore, in our multivariate logistic regression, FB was the only risk factor for elevated pSDC-1, which surpassed the CPB duration and PCT.

Another important result was that, although the progressive AKI incidence was significantly higher in the high pSDC-1 subgroup than in the low pSDC-1 subgroup in both FO and non-FO groups, the odds ratio was higher in the FO group. This means that the elevated pSDC-1 combined with fluid overload may further aggravate AKI. It is not new that fluid overload leads to AKI progression and worse outcomes. The present study showed that the number of patients with fluid overload in the progressive AKI group was significantly higher (58.1 vs. 22.6%, *p* < 0.001). Our previous study found that both excessively negative and positive accumulative 48-h FBs after cardiac surgery increased the risk of AKI progression (33). Patients with fluid overload tend to have more severe endothelial dysfunction with shedding of the glycocalyx and subsequent capillary leakage (13). This may cause intravascular hypovolemia, thereby prompting the need for more fluid administration. Furthermore, there has been an interconnected relationship between AKI and FO (34). It is very important to understand this vicious circle between AKI and fluid and endothelial dysfunction.

We chose 5% as the definition of fluid overload because fluid administration is relatively strict in our center. Our previous study showed that the cumulative fluid balance within 24 h after surgery was 0.7% (−0.4 to 1.9%) in all surgery patients (33). In the present study, the FB from surgery to AKI diagnosis was 3.4 ± 2.8%, and there were only 1/3 of patients with FB > 5%. Furthermore, studies have demonstrated that FB > 10% is associated with adverse outcomes, the variables were more severe by receiving RRT and the adverse event referred to mortality (35, 36), while our study was AKI progression.

Shed SDC-1 in blood can be filtered by glomeruli and detected in urine (37). However, in our present study, only the quartiles of pSDC-1 concentration showed a graded association with the incidence of progressive AKI, ranging from 5.0 to 52.4%, while this trend was not observed for uSDC-1. It's very interesting that the difference between plasma and urine SDC-1 seems to predict progressive AKI (as in Figure 2, pSDC - uSDC <0 in Q1 and Q2, but >0 in Q3 and Q4). This seems likely due to shifting relationship between the gradient for filtration and the GFR, as with mild AKI, GFR is intact and high pSDC is filtered and excreted, but with loss of GFR at high pSDC, the uSDC drops as it is no longer filtered or excreted.

Our study has some limitations. First, although it was a prospective study, it was also a single-center study with small samples, which needs further confirmation. Second, the samples were evaluated at the time of AKI diagnosis which was highly variable. Although we tried the best to make the diagnosis as early as possible (e.g., the urine output was recorded every 6 h after surgery, and the SCr was monitored at least once every day), it is still hard to diagnosis at the earliest time for each patient. Third, we adjusted the SCr for fluid balance in all samples, and fluid overload may not the only reason for artifactually low creatinines after cardiac surgery. In some centers and some patients ultrafiltration is performed during CPB, and this results in reduced creatinine. Fourth, although ELISA tests have previously been used to measure syndecan-1 concentration in urine samples (13, 24), the measurement of urinary glycocalyx is still not very clear, thereby prompting more studies for more accurate measurement modalities.

## Conclusion

Elevated pSDC-1 concentration measured at AKI diagnosis was associated with progressive AKI after cardiac surgery, and pSDC-1 can predict progressive AKI well. The interaction between pSDC-1 and FO may further aggravate AKI. This may represent a potential biomarker to identify the risk of AKI progression after cardiac surgery and highlights the risk of fluid overload in the treatment of CSA-AKI.

## Data Availability Statement

The raw data supporting the conclusions of this article will be made available by the authors, without undue reservation.

## Ethics Statement

The study was conducted ethically in accordance with the World Medical Association Declaration of Helsinki, and was approved by the Ethical Committee of Zhongshan Hospital affiliated to Fudan University (Approval No. B2018-175, Principal Investigator: XD).

## Author Contributions

JX did the study design, collected samples, extracted data, performed the analysis and drafted the manuscript. WJ and YL extracted data, performed the analysis, and revised the manuscript. JT, XX, and XD conceived the idea, did the study design and participated in manuscript revision. XG and JH performed the experiment and helped to generate figures. HL and XC collected samples and data and performed the literature research. JH, BS, YF, and YW helped to do the study design and revise the manuscript. CW, GT, and ZL provided patients and participated in manuscript revision. All authors contributed to the article and approved the submitted version.

## Conflict of Interest

The authors declare that the research was conducted in the absence of any commercial or financial relationships that could be construed as a potential conflict of interest.

## Publisher's Note

All claims expressed in this article are solely those of the authors and do not necessarily represent those of their affiliated organizations, or those of the publisher, the editors and the reviewers. Any product that may be evaluated in this article, or claim that may be made by its manufacturer, is not guaranteed or endorsed by the publisher.
